# Recent advances in novel tumor immunotherapy strategies based on regulating the tumor microenvironment and immune checkpoints

**DOI:** 10.3389/fimmu.2025.1529403

**Published:** 2025-06-18

**Authors:** Hanhui Jing, Yan Gao, Zongsheng Sun, Shanglong Liu

**Affiliations:** Department of Gastrointestinal Surgery, the Affiliated Hospital of Qingdao University, Qingdao, Shandong, China

**Keywords:** immune checkpoint, tumor microenvironment, cancer immunotherapy, immune cells, immunotherapy resistance

## Abstract

Tumor immunotherapy, a novel and rapidly progressing cancer treatment, has experienced remarkable advancements over recent years. It focuses on augmenting the patient’s immune defenses and remodeling the immune microenvironment (IME) of tumors, rather than directly targeting malignant cells. The efficacy of immunotherapy relies substantially on multiple components within the tumor microenvironment (TME), extending beyond adaptive immunity alone. Immune cells within the TME play critical roles in both promoting immune surveillance and facilitating immune evasion. This complexity emphasizes the importance of immune checkpoint regulation in immunotherapeutic interventions. Therapeutically targeting specific immune cell subsets and metabolic pathways in combination treatments can transform an immunosuppressive TME into one that is immunologically activated, facilitating enhanced immune cell infiltration and consequently improving immunotherapy efficacy. Nevertheless, comprehensive research remains necessary to fully elucidate the mechanisms underlying TME interactions and immune checkpoint regulation, ultimately enabling more effective immunotherapeutic approaches.

## Introduction

Over the past decade, tumor immunotherapy has rapidly evolved into a promising therapeutic modality. Rather than directly attacking tumor cells, immunotherapy leverages the body’s immune response by enhancing innate defenses and reshaping the IME. Its primary objective is to potentiate natural anti-tumor immunity through increased infiltration of adaptive and innate immune cells into the TME. The formation of a favorable IME and enhanced immune responsiveness holds substantial clinical potential for predicting therapeutic outcomes and exploring new treatment avenues. Immunotherapy is associated with fewer adverse effects compared to conventional chemoradiotherapy. Targeting immune checkpoints, a cornerstone of immunotherapy, exhibits synergistic effects when combined with chemotherapy, radiotherapy, or targeted therapies. The contemporary paradigm of advanced cancer management has progressively transitioned from chemotherapy and targeted therapies toward immunotherapy, increasingly integrating neoadjuvant and adjuvant treatment modalities.

Immune checkpoints represent crucial inhibitory molecules within the immune system, predominantly expressed on immune and tumor cell surfaces. Upon receptor engagement, these molecules inhibit immune cell activation or promote immune exhaustion, exerting immunosuppressive effects. Under physiological conditions, immune checkpoints are essential for maintaining immune tolerance and preventing autoimmunity. Recently, extensive studies have primarily focused on immune checkpoints, both pivotal in mediating tumor immune evasion. Continuing research efforts have identified additional checkpoints. Immune checkpoint blockades (ICBs), mainly antibodies targeting programmed death protein-1 (*PD-1*), programmed death ligand-1 (PD-L1), and cytotoxic T lymphocyte-associated antigen-4 (*CTLA-4*), represent the primary immunotherapeutic strategy currently employed.

Presently, a significant limitation of immunotherapy, particularly ICB, is its restricted therapeutic response observed in subsets of cancer patients. Response rates vary widely across distinct cancer types and among patients diagnosed with identical malignancies, considerably restricting ICB’s broader clinical utility. Differential responses to immunotherapy, including immune checkpoint inhibitors (ICIS), are predominantly attributed to variations in tumor IMEs across cancer types and subtypes. Immunosuppressive TMEs inhibit immune effector cells, leading to their exhaustion or functional impairment, thus hindering effective tumor eradication. Consequently, exploring novel molecular targets aimed at improving the IME constitutes a key direction in immunotherapy research. The roles and functions of immune cells within tumor contexts are summarized in **Table 1**.

**Table 1:** The role and effect of various immune cells in cancer.

Cell types	Roles in cancer	Effect
Effector T cells	Killing cancer cells by directly identify; Secreting multiple cytokines to induce tumor apoptosis; Transforming into memory T cells for a long time.	Anti-tumor
Regulatory T cells (Tregs)	Inhibits effector T cells and promotes tumor growth and spread	Pro-tumor
NK cells	The release of perforin and granulin leads to apoptosis of cancer cells	Anti-tumor
Dendritic cells	Presents antigens and provides costimulatory signals and adhesion molecules for T cell activation; Produce high levels of the pro-inflammatory cytokines	Anti-tumor
M1-polarized macrophages	Pro-inflammatory cytokines are produced and Th1 is activated to kill tumor cells; Inhibits the formation of tumor neovascularization	Anti-tumor
M2-polarized macrophages	Secretion of TGF-β,IL-10 cytokines impair the immune response	Pro-tumor
N1-polarized neutrophils	Release cytotoxins, secrete cytokines, and promote apoptosis of tumor cells	Anti-tumor
N2-polarized neutrophils	Supports angiogenesis and secretes immunosuppressive factors such as ROS	Pro-tumor
Myeloid-derived suppressor cells (MDSCs)	Inhibits immune cells, remodels the extracellular matrix, and promotes immune escape	Pro-tumor
B cells	On the one hand, tumor cells are cleared through antibody-mediated cytotoxicity, and on the other hand, the immune microenvironment is regulated to promote tumor growth and metastasis	Anti-tumor & Pro-tumor

## TME and immunotherapy

**Figure 1:**
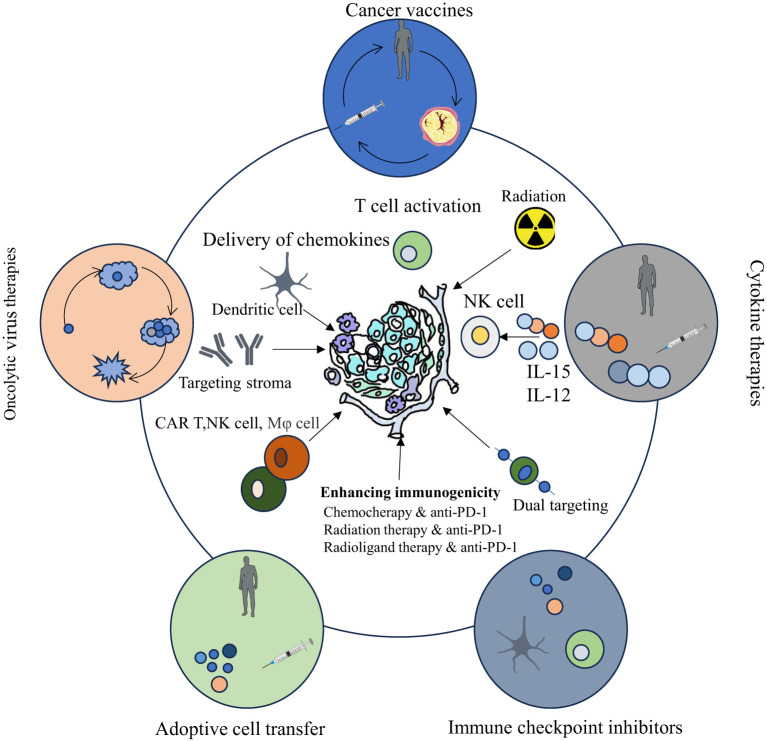
Cancer immunotherapy categories (oncolytic viruses, vaccines, cytokines, cell transfer, checkpoint inhibitors) have evolved, showing clinical promise, with their principles and cellular/molecular underpinnings depicted.

The TME encompasses the local surroundings in which tumor cells exist (1–4). Rapid proliferation of tumor cells accompanied by underdeveloped vasculature results in insufficient oxygen delivery, creating a hypoxic environment within tumor tissue (5, 6). Additionally, tumor cells preferentially generate energy through aerobic glycolysis, causing lactic acid buildup (7–9). Vascular anomalies and metabolic dysfunction trigger cascades of signaling pathways that foster the establishment of an immunosuppressive TME (5). Tumor-infiltrating immune cells (TIICs) critically influence cancer cell activity within this microenvironment. These cells exhibit considerable heterogeneity and plasticity, exerting dual roles that either suppress or promote tumor growth. TIICs primarily encompass cancer-associated fibroblasts (CAFs), tumor-associated macrophages (TAMs), T lymphocytes, B lymphocytes, dendritic cells (DCs), neutrophils, natural killer (NK) cells, and myeloid-derived suppressor cells (MDSCs) (10). Microscopically, the TME is distinguished by pronounced fibrosis, limited vascularization, extensive interstitial fibrosis, abundant CAFs, and marked infiltration of immune cells with pro-inflammatory and tumor-promoting characteristics. Moreover, the immunosuppressive nature of the TME represents a defining feature of malignancies and constitutes a critical site for interactions between tumor cells and host immunity (11, 12). Therefore, modulation of immune cells within the TME to regulate anti-tumor responses has increasingly become a research priority. ICB therapy, representing a major recent advance in tumor immunotherapy, has exhibited notable effectiveness against various cancers **Figure 1**. Emerging technologies and novel research paradigms promise continued improvements in TME-focused immunotherapeutic strategies. Relevant points are summarized in **Table 2**.

**Table 2:** Therapeutic targets that focus on the tumor-associated immune and stromal compartments, either investigated in interventional clinical trials or approved by the FDA.

Classification	Target	Tumor type	Phase/Status	Treatment	Clinical Trials.gov Identifier	Study Start
TAMs	CSF1R	Colorectal cancer and pancreatic ductal adenocarcinoma	Phase 1	Pexidartinib with Anti-PDL1 Antibody	NCT02777710	2016-06
	CCL2	Metastatic Castrate-Resistant Prostate Cancer	Phase 2	Monotherapy	NCT00992186	2009-09
	CCR2	Pancreatic Cancer	Phase 1	Monotherapy	NCT03851237	2019-01-02
	CD40	Locally Advanced Pancreas Cancer	Phase 1	Mitazalimab	NCT06205849	2024-06-25
	SIRPα	Advanced Solid and Hematologic Cancers	Phase 1	CC-95251	NCT03783403	2019-03-01
DCs	GM-CSF	Metastatic Breast Cancer	Phase 2	Herceptin	NCT00429104	2002-08
	FLT3L	Metastatic Colorectal Cancer	Phase 1	Monotherapy	NCT00003431	1998-06
Immune checkpoint blockade	CTLA-4	Advanced Ovarian Cancer Advanced Solid Tumor	Phase 3 Phase 1/2	PD-1/​CTLA-4 Antibody	NCT06542549 NCT03179007	2024-10-01 2017-06-07
	LAG3	Advanced Solid Tumor Malignancies or Lymphomas	Phase 1	Sym022	NCT03489369	2018-05-08
	TIM-3	Advanced Solid Tumor Malignancies or Lymphomas	Phase 1	Sym023	NCT03489343	2018-05-24
	TIGIT	Advanced Tumours	Phase 1	PM1009	NCT05607563	2022-11-21
CAFs	CXCR4	Multiple Myeloma	Phase 1/2	Monotherapy	NCT01010880	2008-10
	FGFR	Solid tumors	Phase 2	Pemigatinib	NCT04003623	2019-10-31

(Data was collected from http://clinicaltrials.gov and accessed in April 2025).

## Targeted TME therapy

Tumor-infiltrating lymphocytes (TILs) comprise diverse lymphocyte subsets predominantly residing within the TME. These cells primarily include T cells, B cells, NK cells, DCs, macrophages, and MDSCs, with T cells being most abundant. *CD4+* T cells mainly differentiate into helper T cells (Th cells) and regulatory T cells (Tregs) (13–15). Th cells further differentiate into specific subsets such as Th1 and Th2 cells, which typically release various inflammatory cytokines to enhance the activity of immune cells. T cells play a pivotal role in orchestrating anti-tumor immune responses. However, infiltrating *CD8+* T cells exhibit elevated expression of co-inhibitory molecules, coupled with reduced proliferation markers like *Ki-67*, indicative of functional exhaustion and impaired effector capabilities. An acidic microenvironment further diminishes T-cell-derived pro-inflammatory cytokines, while increasing *CTLA-4* expression. Consequently, infiltrating T cells become increasingly susceptible to inhibitory signals (5, 16, 17). Hypoxic conditions within tumors lead to diminished *CD4+* T cell populations and elevated expression of immunoregulatory factors, such as *VEGF* and *IDO*. These molecules inhibit antigen-specific immune responses and decrease *IFN-γ* production from CTLs (18–20).

Alterations in metabolism constitute key hallmarks of tumors. Tumor cells modify metabolic pathways and nutrient uptake to sustain rapid proliferation. In the immunosuppressive TME, tumor cells limit nutrient availability required for T cell activation and generate abundant lactic acid, resulting in nutrient scarcity and metabolic waste accumulation. These conditions prompt phenotypic and functional shifts in TIL populations (21, 22). In the hypoxic and nutrient-deficient TME, tumor cells preferentially acquire and rapidly consume glucose, favoring glycolysis over oxidative phosphorylation (OXPHOS) due to its metabolic advantages. This intense glycolytic activity results in substantial lactic acid accumulation (23, 24). Inhibiting lactate production using inhibitors of lactate transporters can enhance *IL-2* and *IFN-γ* secretion in T cells and promote T cell activation. Alterations in tumor lipid metabolism also significantly affect T cell activity. Cholesterol and its derivatives critically regulate T lymphocyte function, including chemotaxis, cell cycle, and effector functions (25, 26). Interventions targeting membrane cholesterol represent a potential strategy for modulating T cell activation. Studies have shown that genetic knockout or pharmacological inhibition of ACAT1 in *CD8+* T cells suppresses intracellular cholesterol esterification. Consequently, increased free cholesterol translocates to the cell membrane, raising membrane cholesterol levels and enhancing *CD8+* T cell activation (27, 28). In preclinical melanoma and lung cancer models, deletion of ACAT1 in *CD8+* T cells significantly suppressed tumor progression and metastasis (29–31). Additionally, attaching liposomes loaded with the ACAT1 inhibitor Avasimibe onto T cell surfaces increases membrane cholesterol content, facilitates rapid T cell receptor clustering, and sustains T cell activation, enhancing their cytotoxic effects against glioblastoma and melanoma. Studies revealed that RORα suppresses genes associated with cholesterol esterification in *CD8+* T cells by inhibiting NF-κB signaling, thereby strengthening *CD8+* T cell-mediated cytotoxic responses (32–34). Elevated cholesterol levels in *CD8+* T cells induce ER stress, activating the ER stress-related protein XBP1. XBP1, functioning as a transcription factor, enhances expression of inhibitory molecules, resulting in functional exhaustion and suppression of *CD8+* T cells, and ultimately promoting tumor progression (35–37).

Regulatory B cells are crucial to immune regulation, suppressing inflammatory responses primarily through *IL-10* secretion. Recent studies have linked cholesterol metabolism to the anti-inflammatory functions of B cells. Specifically, the synthesis of GGPP, a cholesterol pathway metabolite, is essential for inducing *IL-10* production. This process suppresses the Th1 response and limits overall immune reactivity, highlighting cholesterol metabolism as a pivotal pathway in *IL-10* production and B cell regulation (38–40).

In recent years, non-coding RNAs (miRNA, lncRNA, and circRNA) have been identified as critical for the development of various cancers, and their aberrant expression serves as diagnostic and therapeutic markers (41, 42). miRNAs can bind directly to the 3’-UTR or target other genes to regulate PD-L1 expression. Cortez et al. found that in NSCLC, wild-type P53-induced miR-34 directly binds to the 3′-UTR of PD-L1 to inhibit PD-L1 mRNA expression, representing a potential therapeutic strategy by modulating the tumor immune escape mechanism via the p53/miR-34/PD-L1 axis (43). Xia et al. reported that LINC01140 overexpression protects PD-L1 mRNA from miRNA-mediated suppression, facilitating immune evasion in lung cancer cells (44). Additionally, exosomes secreted by cells are rich in miRNA, mRNA, and functional proteins, mediating cell-to-cell signaling within the TME (45). In gastric cancer, increased PD-L1 expression through EV-mediated miR-675-3p promotes immune evasion by cancer cells (46).

## CAFs

Fibroblasts are critical multifunctional cells within connective tissues, responsible for synthesizing extracellular matrix and basement membrane components, modulating immune responses, influencing epithelial differentiation, and sustaining tissue integrity (47). Tumor cells induce activation and differentiation of fibroblasts into CAFs through direct intercellular contact or secretion of soluble signaling factors. CAFs prominently secrete proteins of the *TGF-β* family, particularly *TGF-β1 (* 48). Additionally, CAFs suppress *CD8+* T cell activity by expressing immune checkpoint ligands, thereby facilitating tumor immune evasion (49, 50). Therapeutic strategies targeting CAFs enhance the anti-tumor activities of cytotoxic T lymphocytes and NK cells while reducing regulatory Treg and MDSC populations (51, 52). Current research mainly focuses on CAF-targeted approaches by inhibiting their secreted cytokines and chemokines. For instance, combining *TGF-β* pathway inhibitors with anti-*PD-1* antibodies disrupts *TGF-β* signaling, increases T cell infiltration, and augments anti-tumor immunity. Khalili et al. demonstrated that melanoma-derived *IL-1α* and *IL-1β* increase CAF density, whereas cytokine neutralization mitigates CAF-mediated suppression of T cell activation (53). Multiple studies confirm the significant role of CAFs in resistance to immunotherapy, indicating CAF interactions with diverse immune cells as promising therapeutic intervention targets. Several clinical trials involving CAF-targeted drugs combined with existing therapies are underway (4, 49). Despite progress, CAF heterogeneity has hindered therapeutic efficacy, potentially causing off-target effects (54–56).

## MDSCs

MDSCs are a heterogeneous population of immature myeloid cells from bone marrow, including granulocytes and monocytes. Their primary feature is potent suppression of T cell responses, positioning them as key mediators of tumor-induced immunosuppression (57, 58). Activated MDSCs release immunosuppressive factors, which inhibit CTLs, NK cells, and their subsets, promoting tumor immune evasion and resistance to immunotherapy (59, 60). High-fat diets and obesity can enhance MDSC accumulation in tumor-bearing mice (61). *CYP27A1* synthesizes *27-HC*, a cholesterol metabolite positively correlated with poor prognosis. Studies have shown *27-HC* promotes M-MDSC differentiation and proliferation, facilitating tumor progression by creating an immunosuppressive environment (59, 60). Tumor-derived chemokines recruit MDSCs into primary or metastatic sites in cancers such as breast, gastric, and ovarian tumors (58, 59). Macrophage-derived ApoE in pancreatic cancer binds LDLR on tumor cells, activating the NF-κB pathway and elevating *CXCL1* and *CXCL5* expression (62). *CXCL1* and *CXCL5* recruit M-MDSCs, mediating immunosuppression by inhibiting *CD8+* T cell infiltration, thereby promoting tumor progression (63). In ovarian cancer (OC) and melanoma, ApoE binds LRP8 on MDSCs, enhancing anti-tumor immunity (64, 65). The *LXR*/ApoE axis influences MDSC survival, and *LXR* agonists (*RGX-104/GW3965*) have demonstrated efficacy in mouse models, significantly reducing tumor growth and metastasis by inducing MDSC apoptosis (66–68). *LXR* agonists also potentiate *PD-1* blockade efficacy by targeting TAMs and MDSCs. Currently, the *LXR* agonist *RGX-104/GW3965* is undergoing clinical trials for stage I solid tumors (NCT02922764) to investigate MDSC-mediated immunosuppression mechanisms and therapeutic potential (68, 69).

Tregs are immunosuppressive cells that inhibit anti-tumor immune responses. They suppress effector T cells via *CTLA-4* expression and cytokines (70). *IL-10* primarily mediates Treg immunosuppression by inhibiting pro-inflammatory cytokines from monocytes and macrophages, reducing *IL-12* synthesis, and hindering Th1 differentiation. Neutralizing antibodies against *IL-10* can block Treg-mediated effector T cell suppression (71, 72). Tregs highly express *CD25*, enabling them to compete effectively for *IL-2*, resulting in effector T cell depletion and apoptosis (73).

*TGF-β* critically mediates immunosuppression by inhibiting effector T cell activation and promoting the differentiation of Tregs and Th17 cells (74). Tregs maintain immune tolerance partly by promoting activation of latent *TGF-β1 (* 74). Additionally, Tregs express integrin αvβ8, activating *TGF-β* and mediating immunosuppression through cytotoxic mechanisms. Granzyme B, a serine protease delivered into target cells via perforin, initiates caspase-3-dependent apoptosis. By secreting granzyme B, Tregs induce apoptosis of effector T cells, thus modulating immune responses. The expression of granzyme B in Tregs relies significantly on *TCR/CD28* signaling through activation of the *PI3K-mTOR* pathway (75). *CCL1* activates Tregs by increasing surface expression of *CCR8*, and interaction between *CCL1* and *CCR8* induces Stat3-dependent Granzyme B expression, enhancing Treg inhibitory activity. Tregs also mediate immunosuppressive responses by altering cell metabolism. They require more glucose than effector T cells to execute immunosuppressive functions, leading to effector T cell exhaustion due to competitive glucose consumption (76, 77).

cAMP inhibits T cell activation and function. In Tregs, *FOXP3* increases intracellular cAMP by enhancing *AC9* expression through suppression of miR-142-3p and *PDE3b*. Subsequently, Tregs directly transfer cAMP to effector T cells through cell-to-cell contact, impairing their proliferation and reducing *IL-2* secretion. Furthermore, Treg interactions with DCs elevate DC cAMP levels, downregulating expression of co-stimulatory molecules CD80*/CD86*. Surface-expressed *CTLA-4* on Tregs further suppresses CD80*/CD86* expression, impairing DC-mediated T cell activation. Tregs also secrete *IL-10*, inhibiting DC maturation and reducing their antigen-presenting capability (78–81).

*CD70*, a TNF family member expressed on dendritic and thymic medullary epithelial cells, enhances cytotoxic T cell function. Tregs down-regulate DC membrane *CD70* expression via a *CD27*-dependent mechanism, thus impairing DC function. Selective depletion of Tregs from the TME improves anti-tumor immune responses. Current immunotherapies targeting Tregs primarily involve surface molecules overexpressed on Tregs but not conventional T cells. *CD25* (*IL-2*Rα) is the earliest identified Treg marker. Tregs competitively bind *IL-2* through *CD25*, inhibiting effector T cell proliferation and activation. Administration of anti-*CD25* monoclonal antibodies before tumor inoculation significantly suppresses tumor growth in mice and enhances *CD8+* T cell infiltration. Recombinant *IL-2*-diphtheria toxin conjugates selectively remove CD25+ Tregs from cancer patients, enhancing cytotoxic T cell proliferation and cytotoxicity *in vitro*.

*CTLA-4*, highly expressed on Tregs, functions as an immunosuppressive molecule that facilitates tumor cell survival. Tumor-infiltrating *CTLA-4*+ Tregs evade anti-tumor immune responses by dampening effector T cell activities. Anti-*CTLA-4* antibodies enhance anti-tumor effects of *CD4+* and *CD8+* T lymphocytes (82, 83). *CTLA-4* also inhibits glycolytic metabolism in T cells within the TME; therefore, *CTLA-4* blockade enhances glycolysis in Tregs, altering their stability and facilitating activation of *CD8+* TILs *in vivo*, especially in tumors with limited glycolysis (84). Ipilimumab, an FDA-approved anti-*CTLA-4* monoclonal antibody, is currently employed for treating melanoma, and several other cancers. It selectively reduces intratumoral Treg populations via antibody-dependent cellular cytotoxicity (ADCC) mediated by *CD16+* monocytes. Additionally, intratumoral ipilimumab treatment recruits *CD68+CD16+* M1 macrophages, facilitating Treg clearance (85, 86).

**Figure 2:**
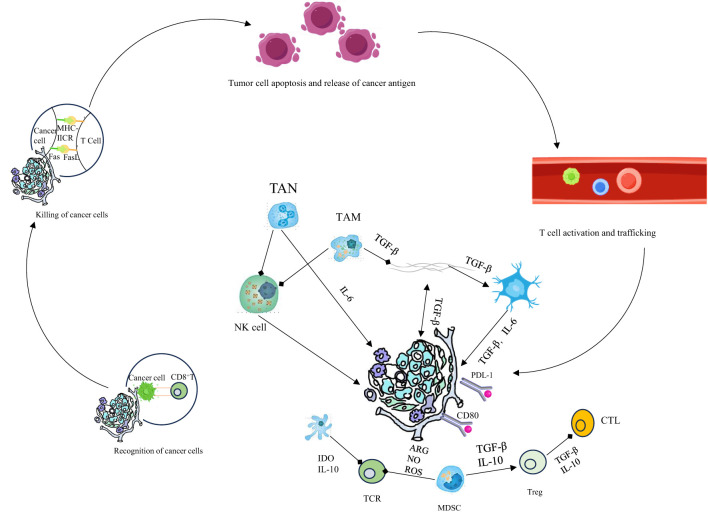
Innate immunity is crucial for the cancer - immunity cycle. Activated by tumors, its cells kill tumor cells directly and prime, expand, and infiltrate tumor - specific T - cells. Therapeutic manipulation of it stimulates antitumor immunity and overcomes immune evasion.

Chemokine receptors CCR4 and *CCR8* are preferentially expressed on Tregs; CCR4 interacts with ligands *CCL1*7 and CCL22. CCR4-positive Tregs secrete increased levels of *IL-10* and IL-35. CCR4 antagonists markedly reduce tumor-infiltrating Treg numbers and enhance responsiveness to sorafenib in murine liver cancer models (87, 88). Moreover, Mogamulizumab, an anti-CCR4 monoclonal antibody, effectively eliminates Tregs through ADCC in adult T-cell leukemia-lymphoma patients, significantly increasing tumor-specific *CD8+* T cells and promoting secretion of *IFN-γ* and *TNF-α*. Fc-optimized anti-*CCR8* antibodies selectively deplete *CCR8*-expressing Tregs within tumors without affecting *CCR8*+ T cells elsewhere, effectively suppressing tumor growth (89, 90). Additionally, anti-*CCR8* treatment induces persistent anti-tumor responses without triggering harmful autoimmune effects. Although several differentially expressed molecules distinguishing tumor-infiltrating Tregs from conventional T cells have been identified, the paucity of Treg-specific targets significantly restricts clinical translation (91–94). Further research is thus required to elucidate Treg-specific expression markers, as well as their development, differentiation, and biological functions within tumors **Figure 2**.

## TAM

Macrophages are crucial components of innate immunity, exhibiting remarkable functional plasticity. Under varying physiological and pathological states, macrophages polarize into either classically activated M1 or alternatively activated M2 phenotypes. M1 macrophages directly eliminate tumor cells and amplify adaptive immunity by upregulating antigen-presenting genes and co-stimulatory molecules. Conversely, M2 macrophages facilitate tumor progression (95–97). Within the TME, tumor-derived signals recruit monocytes and induce their polarization into TAMs, promoting tumor cell proliferation, epithelial-mesenchymal transition (EMT), and suppressing *CD8+* T-cell-mediated anti-tumor effects. Elevated TAM density correlates with enhanced tumor progression and unfavorable prognosis, whereas TAM depletion restores immune functions in the TME, inhibiting tumor growth. TAM-depleting agents include bisphosphonates and inhibitors targeting colony-stimulating factor-1 (*CSF-1*) and its receptor (*CSF-1R*) (98, 99).

Bader et al. demonstrated that clodronate-mediated TAM depletion reduces polyp formation in colon cancer mouse models, down-regulates transcription factors related to carcinogenesis, and modulates intestinal flora, thus inhibiting tumor progression (100, 101). *CSF-1*, a critical growth factor for the monocyte-macrophage lineage, significantly regulates macrophage chemotaxis, survival, proliferation, and differentiation. Many tumors overexpress *CSF-1*, while its receptor *CSF-1R* is broadly expressed on monocytes. Therefore, inhibiting the *CSF-1*/*CSF-1R* pathway effectively depletes TAMs in tumors. *CSF-1R* inhibitors, such as *BLZ945* and *PLX5622*, are widely utilized. Inhibitors targeting monocyte and macrophage recruitment effectively block monocyte/macrophage infiltration into the TME, suppressing tumor progression.

Metabolic regulation also critically influences macrophage polarization. Directly targeting intrinsic macrophage metabolism alters polarization states (5). TAMs in tumors exhibit enhanced glutamine and fatty acid metabolism, essential for maintaining their M2 phenotype. Elevated fatty acid oxidation in macrophages enhances mitochondrial OXPHOS, reactive oxygen species (ROS) production, phosphorylation of tyrosine protein kinase 1, and activation of *STAT6*, thus promoting TAM polarization (102, 103). Tumor-derived metabolites further affect macrophage polarization, impacting tumor progression. Specifically, tumor-derived lactic acid binds the lipid receptor G2A on macrophages, activating *STAT3* and promoting TAM polarization. Depleting macrophage G2A significantly inhibits their polarization toward TAMs. *CD47*, expressed on the surface of tumor cells, binds signal regulatory protein α (*SIRPα*) on macrophages, preventing macrophage-mediated tumor clearance through phagocytosis (104). Inhibiting the *CD47*/*SIRPα* interaction between macrophages and tumor cells has the potential to restore macrophage-driven anti-tumor immune responses mediated by TAMs (105). Combining *CD47*/*SIRPα*-targeted treatments with other therapeutic modalities, including angiogenesis inhibitors and ICIS, can further suppress tumor progression.

Currently, several principal therapeutic agents targeting *CD47*/*SIRPα* include: (1) Hu5F9-G4 monoclonal antibody, a macrophage checkpoint inhibitor targeting *CD47*, promotes tumor cell elimination via macrophage-mediated phagocytosis. Advani et al. demonstrated that combining *Hu5F9-G4* with rituximab effectively enhanced antibody-dependent cellular phagocytosis (ADCP) to treat B-cell non-Hodgkin lymphoma. Clinical trials have confirmed significant therapeutic efficacy of Hu5F9-G4 in aggressive and indolent lymphomas (106, 107). (2) CC-90002, a high-affinity humanized monoclonal antibody against *CD47*, disrupts *CD47*-*SIRPα* interactions. Narla et al. indicated significant dose-dependent anti-tumor effects of CC-90002 (108, 109). (3) ALX148 (Evorpacept), an engineered fusion protein comprising a modified *SIRPα*D1 domain and inactive human *IgG1* Fc, binds *CD47* with high affinity to block interactions with native *SIRPα*. ALX148 promotes innate anti-tumor immunity by increasing macrophage phagocytosis, DC activation, and inflammatory TAM polarization. Combining Evorpacept with anti-*PD-1* or anti-PD-L1 antibodies markedly boosts macrophage phagocytic activity, pro-inflammatory polarization, and DC stimulation, thus potentiating tumor cytotoxicity. Consequently, Evorpacept has emerged as a promising therapeutic candidate targeting *CD47 (* 110). The U.S. Food and Drug Administration (FDA) has approved Evorpacept to treat head and neck squamous cell carcinoma (HNSCC), and HER2-positive gastric or gastroesophageal junction malignancies.

Although TAM depletion strategies inhibit tumor progression, the non-specific effects necessitate further investigation to determine the selective impact on TAM populations and potential collateral effects on beneficial resident macrophages and other immune cells.

## DCs

DCs are professional antigen-presenting cells capable of initiating strong anti-tumor immune responses. Increased infiltration of DCs into tumor tissue correlates positively with improved patient prognosis (111). Studies indicate that patients with higher DC infiltration at tumor margins show lower lymph node metastasis rates and better overall survival compared to those lacking DCs. Therefore, DC-based immunotherapy represents a promising strategy for treating cholangiocarcinoma (CCA) (112). Agents that activate DCs or reverse their immunosuppressive functions enhance both DC and T cell activation. GM-CSF directly promotes DC maturation, activation, and migration (113). *TLR7/TLR8* agonists stimulate NF-κB signaling, promoting secretion of pro-inflammatory cytokines and increasing the expression of co-stimulatory molecules. Imiquimod, a synthetic *TLR7/TLR8* agonist, enhances DC-mediated cytotoxicity and is approved for topical treatment of non-melanoma skin cancers. Clinical trials involving *TLR7/TLR8* agonists (e.g., NCT02574377, NCT02692976) are currently underway (114). Additionally, unmethylated CpG oligodeoxynucleotides, representing major TLR9 agonists, activate human DCs, facilitating Th1-biased immune responses and *CD8+* T cell-mediated anti-tumor immunity. Clinical evaluations combining CpG oligodeoxynucleotides with ICIS are ongoing (NCT02521870, NCT03831295) (115).

DCs bridge innate and adaptive immunity by activating and programming T cells. Studies suggest that cholesterol, hydroxysteroids, and cholesterol transporters influence DC differentiation and maturation. For instance, *27-HC* induces monocyte differentiation into mature DCs, promoting surface expression of characteristic molecules such as MHC-II and CD80, thereby enhancing immune responses (5). Cyclosporin A, a broad-spectrum immunosuppressant, inhibits *27-HC*-induced DC differentiation by interacting with calcineurin, down-regulating specific DC markers (116). The absence of ApoE leads to cholesterol accumulation on DC membranes, enhancing antigen presentation through increased aggregation of MHC-II molecules, thus strengthening *CD4+* T cell-mediated immune responses. Conversely, oxidized lipids impair DC cross-presentation in cancer by promoting accumulation of triacylglycerols, and fatty acids in DCs, reducing MHC-I expression and exogenous antigen presentation (5). Additionally, liver X receptor (*LXR*) activation impairs DC migration to lymphoid organs by suppressing CCR7 expression, promoting tumor immune escape.

Several clinical trials have explored DC vaccines, involving the isolation, expansion, and *in vitro* manipulation of autologous DCs for re-injection into patients. These studies primarily targeted immunogenic cancers, such as prostate cancer and glioblastoma, confirming the safety and clinical efficacy of DC-based vaccines in stimulating NK cells and *CD8+* T cell responses (117). Currently, sipuleucel-T (Provenge), an autologous APC vaccine loaded with prostate-specific antigen-GM-CSF fusion proteins, represents the only clinically approved APC-based vaccine. Clinical trials demonstrated that sipuleucel-T extends median overall survival by approximately four months in prostate cancer patients. DC-based therapies have the potential to enhance current cancer treatments; however, developing optimal vaccine strategies requires deeper understanding of DC biology and function (118). Preclinical studies indicate that DC-based anti-tumor immunotherapy holds considerable promise, warranting further clinical validation.

Neutrophils represent an important immune cell population within the TME. Increased proportions of tumor-associated neutrophils (TANs) occur frequently in various solid tumors, exhibiting similar pro-tumor activities as PMN-MDSCs (119). Tumor-derived *22-HC* recruits Tumor-derived *22-HC* recruits TANs through *CXCR2* signaling, promoting angiogenesis, immunosuppression, and tumor growth. Additionally, hypoxia-inducible factor-1α (HIF-1α) induces *24-HC* synthesis via CYP46A1, facilitating anti-inflammatory neutrophil infiltration and angiogenesis in pancreatic neuroendocrine tumors (120). TANs mediate immunosuppression via PD-L1, impaired antigen presentation, ROS, and related pathways, representing emerging therapeutic targets and prognostic indicators (121, 122). The neutrophil-to-lymphocyte ratio (NLR) is a potential biomarker for tumor prognosis. TAN infiltration closely associates with tumor progression, and quantitative analysis of TANs, Tregs, and TAMs interactions can predict cancer patient outcomes (123).

Targeting TANs with small-molecule inhibitors or neutralizing antibodies is a promising therapeutic strategy. Studies indicate down-regulation of methyltransferase-like 3 (*METTL3*) elevates IL-8 expression, enhancing N2 TAN recruitment. IL-8 antagonists eliminate N2 TAN accumulation, significantly delaying tumor growth in mice (124). *CXCR1/2* inhibitors can prevent immunosuppressive neutrophil recruitment, enhancing *PD-1* therapy efficacy and treatment response rates (119). TANs also inhibit *CD8+* T cell cytotoxicity via *JAG2* signaling. Blocking the Notch pathway with gamma-secretase inhibitor LY3039478 and anti-*JAG2* antibodies delays tumor growth and improves *CD8+* T cell cytotoxicity. Additionally, TAN-secreted IL-17a promotes gastric cancer EMT through *JAK2/STAT3* signaling. Neutralizing IL-17a or blocking JAK2/STAT3 signaling with inhibitor AG490 reduces TAN-mediated tumor migration and invasion (125).

TANs demonstrate high plasticity and heterogeneity, necessitating further research into their characteristics. Current studies employing single-cell sequencing investigate TAN polarization reprogramming to identify new immunotherapy targets. Tumor cell response to immunotherapy depends not only on intrinsic genetic reprogramming but also on the complex interactions and cytokine/chemokine regulation within the TME (121), Understanding TAN-TME molecular interactions and signaling pathways presents new avenues for targeted tumor immunotherapy, reshaping the TME and hindering tumor cell colonization, growth, and invasion (126). Combined therapeutic strategies targeting TANs, tumor cells, and TME components may enhance tumor immunotherapy outcomes.

NK cells, innate lymphoid cells, possess intrinsic capacity to recognize and eliminate malignant cells independently of prior sensitization. NK cells exhibit potent tumoricidal activity, promoting apoptosis via secretion of perforin, cytotoxic molecules, and TNF. The activating receptor natural killer group 2D (NKG2D), predominantly found on NK cells, mediates tumor recognition and cytotoxicity (127). Cholesterol accumulation in NK cells promotes their activation and enhances their cytotoxic function, significantly influencing cancer progression, notably in hepatocellular carcinoma. Additionally, activation of *LXR* signaling in multiple myeloma cells elevates NK-cell-mediated cytotoxicity by upregulating NKG2D ligands, including MICA and MICB (128).

*PD-1*, conventionally recognized as an exhaustion marker on T cells, is also expressed on NK cells. Tumor-derived exosomal circUHRF1 from hepatocellular carcinoma enhances *PD-1* expression in NK cells, thus weakening their anti-tumor capacity (129). Similarly, in gastrointestinal malignancies, elevated *PD-1* levels on NK cells impair their cytotoxic activities due to PD-L1 binding; disrupting *PD-1*/PD-L1 interactions restores NK cell functions. Additionally, TIM-3 is another marker of NK cell exhaustion; dual TIM-3 and *PD-1*-positive NK cells exhibit reduced secretion of *IFN-γ* and granzyme B, limiting their cytotoxic effectiveness (130). NK cell effector functions in tumors are compromised by inhibitory TME interactions.

Clinical approaches enhancing NK cell function have yielded promising outcomes. A phase III/IVA trial in head and neck cancer demonstrated that *PD-1*+ NK cell enrichment induced by anti-EGFR antibody cetuximab predicts favorable prognosis. Subsequent anti-*PD-1* antibody nivolumab administration significantly enhanced cetuximab-induced NK cell activity. In colon cancer, TIGIT blockade prevents NK cell exhaustion, thereby augmenting NK-driven anti-tumor responses and improving T-cell-mediated immunity in an NK-dependent manner. Moreover, re-administration of anti-PD-L1 antibodies enhances persistent immune memory (131). Monalizumab, a monoclonal antibody targeting NKG2A, enhances NK cell cytotoxicity and restores *CD8+* T cell functions. Phase II clinical trials combining monalizumab with cetuximab in head and neck carcinoma showed an objective response rate of 31%. *TGF-β*, an important immunosuppressive molecule that induces NKG2A expression, is also an emerging therapeutic target. Inhibitors such as galunisertib block *TGF-β*, thereby augmenting NK and T cell cytotoxicity and improving outcomes from anti-*PD-1*/PD-L1 therapies (132). Future developments will likely increase NK-targeted therapies, offering personalized treatment strategies based on tumor-specific characteristics.

## Tumor immunotherapy

Immune checkpoint inhibition (ICI): Upon activation, T lymphocytes involved in anti-tumor immunity up-regulate various inhibitory receptors. These receptors bind ligands highly expressed on tumor cells, suppress immune responses, and weaken anti-tumor immunity. These negative regulatory mechanisms of immune activation are termed immune checkpoints. ICI has emerged as a major area of immunotherapy research. Among extensively studied immune checkpoints are *CTLA-4* and *PD-1*, co-inhibitory receptors expressed by T cells that negatively regulate their function (133). Tumor cells inhibit T cell-mediated immunity primarily by expressing high levels of checkpoint ligands. Immunotherapy strategies employ monoclonal antibodies targeting these checkpoints to enhance endogenous anti-tumor responses. Numerous studies have demonstrated the effectiveness of ICIs in reversing tumor-induced immunosuppression. Currently, *PD-1*/PD-L1 and *CTLA-4* inhibitors represent the most actively investigated checkpoint inhibitors. Additionally, CD40, a co-stimulatory receptor on APCs, has emerged as another promising immunotherapy target. Several CD40 agonists are undergoing clinical trials in oncology and immune disorders. ICIs have shown therapeutic success in various malignancies, including melanoma and hepatocellular carcinoma. Response rates to ICIs correlate closely with tumor-specific genetic profiles, particularly DNA mismatch repair deficiency (dMMR) and microsatellite instability-high (MSI-H) status (134). In 2017, the FDA approved pembrolizumab and nivolumab specifically for MSI-H/dMMR CRC. The Phase III clinical trial KEYNOTE-177, involving 307 treatment-naive metastatic CRC patients with MSI-H/dMMR, randomized patients 1:1 to pembrolizumab (200 mg every 3 weeks) or standard chemotherapy. Median progression-free survival (PFS) improved significantly to 16.5 months with pembrolizumab versus 8.2 months with chemotherapy. At 24-month follow-up, the mean survival duration was 13.7 months for pembrolizumab-treated patients compared to 10.8 months for chemotherapy recipients. Adverse event incidence rates were comparable, at 97% (149/153) for pembrolizumab and 99% (142/143) for chemotherapy (135). Pembrolizumab has also entered Phase II clinical studies targeting *PD-1* in CCA, significantly improving overall survival (OS) and objective response rates (ORR) in patients harboring mismatch repair defects. FDA-approved ICIs, including pembrolizumab, nivolumab, durvalumab, atezolizumab, and avelumab, have demonstrated efficacy in various solid tumors (**Table 3**). Emerging checkpoint inhibitors targeting molecules such as TIGIT, TIM-3, and inhibitory ligands (B7-H3, B7-H4, B7-H5) are currently being intensively studied for solid tumor therapy (136).

**Table 3:** Selected clinical trials for tumor therapy.

Classification	Cancer types	Clinical trials	Phase	Status
Dendritic cell vaccines for cancer immunotherapy	Prostate cancer	NCT00779402	Phase 3	Completed
	Colorectal caner	NCT02503150	Phase 3	Unknown
	Kidney cancer	NCT05127824	Phase 2	Recruiting
	Breast cancer	NCT00266110	Phase 2	Completed
	Melanoma	NCT01876212	Phase 2	Completed
Macrophage-targeted immunotherapies	Solid tumor	NCT01204996 NCT00537368	Phase 1 Phase 1	Completed Completed
	Pancreatic neoplasms	NCT01413022	Phase 1	Completed
	Prostate cancer, Bone Metastases	NCT00757757	Phase 1/2	Terminated
	Advanced solid tumors and lymphomas	NCT02675439	Phase 1	Terminated
	Acute myeloid leukemia	NCT02641002	Phase 1	Terminated
MDSC-based therapeutic strategies	Solid tumors with liver metastases	NCT00094003	Phase 1	Completed
	NSCLC	NCT00752115	Phase 2/3	Completed
	Renal cell carcinoma	NCT04203901	Phase 2	Terminated
	Head and neck cancer	NCT03993353	Phase 2	Recruiting
	Lymphoma	NCT00529438	Phase 1	Completed
Inhibitors of NK cell-associated checkpoints	Solid tumors	NCT05162755	Phase 1	Active, not recruiting
	Urothelial carcinoma	NCT05327530	Phase 2	Active, not recruiting
	Gastric cancer	NCT04933227	Phase 2	Terminated
	Lymphoma or solid tumors	NCT05390528	Phase1/2	Recruiting
Tumor-associated neutrophil (TAN)-targeted cancer therapies	Leukemia	NCT03922477	Phase 1	Terminated
	Cervical cancer	NCT05179239	Phase 3	Recruiting
	Colon cancer	NCT03026140	Phase 2	Recruiting
CAR-NKT therapies	B cell malignancies	NCT03774654 NCT04814004	Phase 1 Phase 1	Recruiting Unknown status
	Neuroblastoma	NCT03294954	Phase 1	Recruiting

## Combination therapy

To enhance the therapeutic efficacy of immunotherapy, combinations of anti-*PD-1*/PD-L1 antibodies with anti-*CTLA-4* antibodies or tyrosine kinase inhibitors (TKIs) have frequently been explored (137). Combination therapies generally exhibit superior efficacy compared to TKI monotherapy. Although both *PD-1* and *CTLA-4* inhibit T cell activation, *CTLA-4* acts primarily during early T cell activation, whereas *PD-1* mainly inhibits activated *CD8+* T cells within the TME (138). Simultaneous inhibition of *CTLA-4* and *PD-1* significantly enhances *CD8+* T cell activation in tumors, exerting synergistic therapeutic effects. The Phase III CheckMate 214 trial demonstrated that nivolumab plus ipilimumab improved PFS, OS, and ORR compared to sunitinib monotherapy in patients with intermediate- or poor-risk advanced renal cell carcinoma (RCC), subsequently leading to FDA approval of this combination therapy (139). In preclinical mouse breast cancer models, ICIs induced *CD8+* T cell activation and vascular normalization in tumors, alleviating immune suppression within the TME and enhancing ICI. This positive feedback between immune activation and vascular normalization provides a rationale for combining immunotherapy strategies. The Phase III JAVELIN Renal 101 trial indicated that combining the anti-PD-L1 antibody avelumab with axitinib extended median PFS by 6.6 months compared to axitinib alone in advanced renal carcinoma patients (140). Similarly, KEYNOTE-426 demonstrated that pembrolizumab (anti-*PD-1* antibody) plus axitinib improved OS, PFS, and ORR versus sunitinib monotherapy. Consequently, the FDA approved these combination therapies in 2019 for advanced renal cancer treatment. In metastatic pancreatic cancer, pembrolizumab combined with CXCR4 inhibitor BL-8040 markedly increased disease control and median OS, associated with elevated *CD8+* T cell infiltration, decreased MDSCs, and stable regulatory Treg levels (141). Furthermore, CXCR4 inhibition enhanced the effectiveness of *PD-1* blockade combined with chemotherapy in advanced pancreatic cancer patients. Animal models of pancreatic cancer liver metastasis demonstrated that gemcitabine combined with *PD-1* blockade improved survival outcomes, increased tumor infiltration of Th1 lymphocytes, and enhanced M1 macrophage activity. Additionally, gemcitabine combined with DC vaccines promoted systemic chemotherapy and T cell-mediated responses. Murine studies further indicated that IL-6 combined with PD-L1 blockade significantly inhibited pancreatic cancer growth. Combining GM-CSF vaccines with *PD-1* blockade notably prolonged survival in pancreatic cancer models (142).

However, combination therapy does not universally benefit all patients and can induce severe adverse reactions. In KEYNOTE-426, diarrhea and hypertension were common with pembrolizumab and axitinib, and liver-related adverse events increased compared with monotherapy, forcing treatment discontinuation in 30.5% of patients. Identifying suitable biomarkers and clarifying drug interactions in combination therapies are thus essential to minimize adverse effects and economic burdens (143).

Stem cell therapy that reprograms the TME provides a novel strategy for overcoming tumor immune escape and enhancing treatment sensitivity by intervening in key aspects such as immunity and vascularization in the TME (127, 144, 145). Macrophages have long been utilized in ACT, but the development of macrophage therapies requires a more cost-effective and durable approach for generating M1 macrophages. Among these approaches, macrophages are engineered to express CAR (CAR-M) (146). Zhang et al. found that induced pluripotent stem cell (iPSC)-derived macrophages (CAR-iMac) have emerged as a promising cellular immunotherapy source (147). In March 2021, the first patient in a phase I multicenter clinical trial received CAR-M therapy targeting HER2 to overcome solid tumors (148). Additionally, promising results have been achieved in preclinical ACT studies using genetically engineered T-cell receptors (TCRs) and chimeric antigen receptors (CARs). In NSCLC, anti-PD-1/PD-L1 combined with CAR-T cell therapy promotes the restoration of normal immune recognition and maintenance of immune system homeostasis (149). Fang et al. reported that PD-1-meso CAR-T cells were effective and safe for advanced ovarian cancer, rapidly improving the TME without obvious adverse reactions (150). However, the long-term efficacy of CAR-T cells remains uncertain in most clinical studies, even for leukemia. Nevertheless, CRISPR/Cas9 technology has significantly advanced the understanding of tumor genomics and contributed to cancer immunotherapy. Lu’s team used CRISPR/Cas9-edited PD-1 knockout T cells in patients with advanced NSCLC. The results showed a median PFS of 7.7 weeks, an OS of 42.6 weeks, and stable disease in two patients (151).

Overall, single-agent immunotherapy exhibits limited efficacy, whereas combination therapies effectively transform the TME from immunosuppressive to immuno-activated states and enhance immune cell infiltration. Additional therapeutic targets in the TME, present opportunities for targeted drug development. Combining such strategies with ICIs is potentially beneficial. Personalized treatment strategies based on patient-specific tumor characteristics will improve treatment outcomes and extend patient survival.

## Predictive biomarkers

Predictive biomarkers are critical for population stratification and efficacy assessment, providing an essential pathway for translating basic research into clinical practice. With the advent of single-cell RNA sequencing (scRNA-seq) and mass spectrometry flow cytometry, many additional predictive markers have emerged due to the generation of abundant genetic information. Cancer stem cells (CSCs) significantly contribute to tumor heterogeneity. CSCs can drive tumor growth, promote disease progression, and are associated with distant metastasis and treatment resistance. Fendler et al. identified a small CSC population through single-cell sequencing and evaluated CSC heterogeneity, providing new insights for clinical applications related to tumor drug resistance and CSC-targeted treatments (152). ctDNA has demonstrated associations with clinical response or survival in patients with melanoma, colorectal cancer (CRC), and gastric cancer receiving anti-PD-1 therapy. Another analysis of 18 patients with MSS metastatic CRC identified ctDNA as a biomarker predictive of responses to nivolumab immunotherapy (153). Single-cell multi-omics studies and innovative high-throughput sequencing technologies have opened new avenues for personalized patient treatments. For example, in heterogeneous diseases such as bladder cancer, gene expression models based on multi-omics sequencing can identify patient populations likely to respond well to cytotoxic drugs, enabling precise targeted therapies (154).

Noninvasive imaging modalities (e.g., PET, magnetic resonance imaging (MRI)) can facilitate monitoring of T-cell activation and anticancer T-cell responses (155–158). Radiomics captures features such as tissue morphology, lesion heterogeneity, and changes during continuous imaging throughout treatment or monitoring (159, 160). Studies report a strong correlation between radiomics features and cellular-level heterogeneity indices. Furthermore, PET and MRI can assess T-cell density by detecting energy metabolism-related substances in tumor tissues (161–163). These non-invasive analytical methods allow dynamic observation of patient responsiveness after treatment.

## Summary and prospects

As more combination therapies emerge, involving ICIS, adoptive cell therapy, and chemoradiotherapy or targeted agents, promising outcomes are increasingly evident. However, immunotherapy efficacy requires further improvement. Currently, no reliable predictive indicators for immunotherapy responsiveness exist. Resistance involves complex multifactorial mechanisms, including T cell exhaustion, immunosuppressive cell infiltration, ineffective tumor immune infiltration, and epigenetic factors. Treatment-related adverse reactions present significant clinical challenges. Tumor heterogeneity and dynamic TME interactions account for varied immunotherapy responses and adverse events. Selecting precise targets, identifying suitable patients, and using combination treatments can partly address immunotherapy limitations. Understanding TME impact on immunotherapy is crucial for identifying more effective targets and therapeutic strategies. A deeper understanding of the spatial-temporal heterogeneity within the TME and its interactions with immunotherapy could guide individualized immunotherapy approaches. Concurrently, sensitive and specific biomarker identification will accelerate translating basic research into clinical practice.
